# Worth a thousand interpersonal words: Emoji as affective signals for relationship-oriented digital communication

**DOI:** 10.1371/journal.pone.0221297

**Published:** 2019-08-15

**Authors:** Amanda N. Gesselman, Vivian P. Ta, Justin R. Garcia

**Affiliations:** 1 The Kinsey Institute for Research in Sex, Gender, and Reproduction, Indiana University, Bloomington, IN, United States of America; 2 Department of Psychology, Lake Forest College, Chicago, IL, United States of America; 3 Department of Gender Studies, Indiana University, Bloomington, IN, United States of America; University of Zurich, SWITZERLAND

## Abstract

Computer-mediated communication (CMC) is pervasive in our lives, influencing social interaction including human courtship. To connect with potential partners via CMC, modern relationship-seekers must master faster and shorter methods of communicating self-disclosure and affect. Although CMC can lack crucial sensory information in this context, emojis may provide useful aid. Across two studies, we assessed attitudes toward and frequency of emoji use, and whether signaling affect via emoji use relates to more romantic and sexual opportunities. Our findings suggest that emoji use with potential partners is associated with maintaining connection beyond the first date, and more romantic and sexual interactions over the previous year. This research provides evidence that emojis convey important affective information to potential partners, and are potentially associated with more successful intimate connection. Implications for multiple theoretical models and methodologies are discussed.

## Introduction

With online and app dating services an increasingly common way people meet partners across all demographics of relationship seekers [1], reliance on computer-mediated communication (CMC) has also extended into people’s intimate relationships. As a result, researchers and daters alike need to modify their understandings of human attraction and courtship in the digital age. Although courtship inherently comes with the challenge of accurately assessing the traits of others [2], previous generations of daters had the benefit of meeting a potential date in person, providing the opportunity to observe their body language and voice, engage in touch, see them interact with others, and so on. Today, many people are communicating, meeting partners, and maintaining interpersonal relationships largely through somewhat limited computer-mediated platforms, relying on these platforms to develop and maintain their relationships. This forces many daters to adapt their way of approaching a partner and assessing interest and compatibility, and to attempt to create the building blocks of intimacy in shorter, more frequent, and more emotionally-limited correspondences than we would typically expect in a face-to-face scenario [3]. In this emotionally and sensorily austere context, what tools can daters use to represent themselves and potentially improve their connections?

In two studies, we demonstrate that emojis are one such tool. We examined attitudes toward emoji use in interpersonal contexts, patterns of emoji use, and related ‘success’ in dating contexts. In Study 1, we report on emoji use with potential partners in a large sample of U.S. single adults. We assessed how and why singles use emojis with potential partners, as well as whether their emoji use related to having more romantic and sexual interactions: more first dates and more sexual behavior over the last year. In Study 2, we replicated the core findings from Study 1 and extended them to a wider array of romantic and sexual connection measures. We position our research at the intersection of affect, evolutionary, and social penetration theories, highlighting the many theoretical and social behavioral factors at play when using CMC in dating and relationship contexts.

### Computer-mediated communication

In face-to-face interactions, people often rely on non-verbal behaviors to effectively express emotion. These behaviors communicate both intentional and unintentional signals, and allow for cultivating a bond through shared affect [4]. However, in text-based CMC, non-verbal expression is not as readily communicated. The reduction of expressive and affective information, combined with the inability to use the same nonverbal cues as in face-to-face interactions to accurately decipher intent, results in a challenging environment that requires more effort to achieve effective communication and mutual understanding [5]. These communication challenges can reduce the quality of social interactions [6] and have the potential to result in misunderstandings and lowered likelihood of forming foundational social bonds.

### Communicating with emoticons and emojis

First introduced as a way to provide non-verbal expression in CMC, emoticons—faces made of characters, such as **:-)** and **:(**—became “non-verbal surrogates” in CMC, providing cues that could not otherwise be conveyed [7,8]. People began using emoticons to express affect and to provide an emotional valence advisement for how to interpret the message, to avoid misunderstandings or negative feelings [9]. Recent research analyzing emails provides evidence of the multifaceted nature of emoticons [10]. Senders used emoticons to convey positive feelings or to denote a joke or irony, but also to provide a strength thermometer—either softening a harsh message or emphasizing a positive one. Other inquiries show that emoticons are generally received in these intended ways. For instance, in an experimental study using chat conversations, a reader’s mood was altered either positively or negatively by the respective emoticon [8].

Though several lines of research have addressed the impact of emoticons on communication, emojis—newer, more graphically enhanced descendants of emoticons that include an extensive variety of expressions and object pictographs—are only just starting to be examined. Emojis were introduced to the global market in 2011, when Apple Inc. incorporated 722 emojis into the iPhone keyboard; since then, the number and types of emojis available has rapidly expanded. Emoji use has subsequently exploded in prevalence, with emojis being tweeted more often than the tilde, the number 5, and the hyphen [11].

To date, studies of emojis have been conducted primarily from a marketing standpoint. Researchers have used emoji-based surveys to generate “emotional product profiles” for food and beverage brands to improve marketing efficacy, and concluded that emojis provided a meaningful method of measuring consumer feelings [12]. Similarly, in a study of randomly-sampled tweets about meals, researchers found tweeters were using emojis to express feelings or emotions about their food, leading the researchers to conclude that “emoji… seem to be an easy and intuitive way to express emotions,” (p. 119) [13]. While other techniques are also used to express non-verbals via CMC such as capital letters, punctuation marks, and chronemics [14], the graphical nature of emojis suggest they possess greater ability to convey the nuances of affective communication that are less likely to be conveyed from other CMC techniques or words alone (see Media Richness Theory) [15].

### Emojis, affect, and interpersonal intimacy

We propose that people use emojis to capitalize on evolved social psychological features of human communication, particularly in the domain of courtship. We use *affect* to refer to the biopsychosocial experience of emotions [16]. Non-verbal cues and signals of affect are a cross-cultural feature of human interaction and communication, with the ability to produce and detect these signals necessarily rooted in our evolutionary legacy as a social species [17–19]. Affective communication thus serves an important function in interpersonal relationship development and relationship maintenance. Based on affect theory [20], affective expression allows partners to gauge relationship interest and progress. In general, partners strive to maximize positive affect and minimize negative affect [21], and affect becomes especially important to many foundational aspects of close relationships, including self-disclosure, mutual understanding, and conflict avoidance. Researchers have argued that people are less skilled at interpreting written text because our ability to communicate, especially with regard to affect and emotional valence, specifically evolved for face-to-face verbal and non-verbal communication [17–19]. Because text-based communication can lack much of the sensory information found in face-to-face interactions that promote affective expression and meaningful communication, the character features (e.g., colors, shapes, actions, faces) of emojis may allow communication partners to more readily express affect and introduce it into CMC.

As many romantic and sexual connections are initiated via CMC, emojis may be useful tools in creating the elementary units of intimacy between partners. While the desire for close relationships [17], including romantic and sexual relationships [22], are an evolved feature of human social psychology, the ways in which affect is expressed has implications for the development and stability of dynamic social relationships. According to social penetration theory [23], as relationships advance, interpersonal communication moves from more superficial to more intimate levels. This development occurs primarily through self-disclosure: partners advance to deeper levels of intimacy as they engage in more affective communication with their partner. Engaging in affective self-disclosure may be more challenging through CMC, thus preventing partners from successfully developing shared understanding and advancing to higher levels of intimacy. However, emojis may help supply the socio-emotional affective components of interpersonal communication into CMC, thereby facilitating intimacy and relationship development. Taken together, we argue that emoji use imbues CMC with aspects of expression, emotional valence, and affect that takes advantage of evolved social psychological features of human communication, which in turn can promote the development of intimate relationships.

### Current research

In this research, we investigate the interpersonal aspects of emoji use to examine emojis as tools to connect with potential relationship partners. We posit that overall emoji use is an indicator of affective expression, and is associated with being more emotive across the affect spectrum, which in turn will positively influence romantic and sexual opportunities. In Study 1, we examined attitudes toward emoji use in dating contexts, and whether more frequent emoji use is associated with more interpersonal connections. In Study 2, we replicated and extended Study 2, using a more recent and differently recruited sample, and including additional measures of intimate connection.

It should be noted that individual preferences for and frequency of CMC use differ. Some individuals have a stronger preference to communicate via CMC than do others [24]. Yet, somewhat independent of one’s preference for CMC, some may use CMC more frequently to communicate than other communication modes. These differences may influence the way individuals approach and use CMC-related technologies in everyday interactions, subsequently impacting interaction with intimate partners via CMC. Thus, while not possible in the secondary analyses used in Study 1, we control for both participant CMC preference and frequency in Study 2.

## Study 1

In Study 1, we examined whether emoji/emoticon use was associated with ‘success’ in intimate connections. Using a large national sample of single American adults, we examined emoji/emoticon use with potential partners, motives behind their emoji use with potential partners, and whether their emoji use was associated with going on more dates and having more sexual encounters over the last year. Because emoticons were created and used for years in CMC before the inception of emojis, in Study 1 we asked respondents about both emoji and emoticon use to account for the wider age distribution and variation in smartphone use among participants in this diverse sample.

## Study 1 method

### Participants

Participants were 5,327 single American adults (2,991 women; 2,335 men; 1 identified gender as other). Age ranged from 18–94 years (*M* = 42.03, *SD* = 16.79). Most (86.8%) identified as straight/heterosexual, 9.6% as gay or lesbian, and 3.5% as bisexual. Most (62.2%) identified as White/Caucasian, 18.8% Black/African-American, 15.0% Hispanic/Latino, 5.9% Asian, 2.0% North American Indian/Alaskan Native/Pacific Islander, and 3.3% identified as “other.”

### Procedure

Data were collected as part of the annual *Singles in America* (SIA) study. SIA is sponsored by the online dating company Match; however, participants were *not* recruited or in any way drawn from the Match population or subsidiary sites. Participants were recruited by ResearchNow (Dallas, TX, USA), using independent opt-in Internet research panels for population-based cross-sectional survey. Participants were recruited from these opt-in research panels, with recruitment targeting based on demographic distributions (i.e., age, gender, ethnicity, region, income) reflected in the most recent Current Population Survey conducted by the U.S. Bureau of the Census, and adjusted in real time using inbound click balancing. All data were collected over the Internet. Data access and analysis procedures were approved by Indiana University’s Institutional Review Board. Research panelists received project descriptions and study information prior to viewing the survey. Participants confirmed their interest in taking part in the study via the online portal before being directed to the survey. Inclusion criteria required being at least 18 years old, fluent in English, and having a relationship status of single (i.e., single and not seeing anyone or single but dating casually).

### Measures

SIA includes an extensive collection of demographic characteristics, as well as attitudinal items largely about dating attitudes and behaviors. For the purpose of the current study, we only examined a small subset of items described below.

#### Demographics

Participants reported their gender, age, sexual orientation, and ethnicity. Only age and gender were used in the current analyses. We controlled for these variables because younger people may be more inclined to communicate via CMC, and because prior research has documented gender differences in relationship-oriented traits including emotional intelligence [25]. Due to statistical power, only participants identifying their gender as woman or man were included in the analyses (i.e., one participant was excluded).

#### Emoji/Emoticon use

Participants were asked, “How frequently do you use emojis/emoticons in your text messages to a date?” Response options were “I never use them” (1), “I hardly use them” (2), “I use them regularly, but not in every text” (3), “I use at least one in every text” (4), and “I use more than one in every text” (5).

#### Emoji/Emoticon motives

Participants reported why they use emojis/emoticons in text messages to a dating partner. Motives provided were, “they give my text messages more personality;” “it’s easier for me to express my feelings;” “it’s faster and easier than writing a full message;” and “it’s trendy and other people use it.” Participants responded by checking all that applied.

#### Number of first dates in the last year

Participants reported how many first dates they have gone on in the last year. Response options ranged from 0–20+.

#### Sexual frequency in the last year

Participants reported how often they have had sex—as defined by the participant—in the last year. Responses ranged from 1 (*daily*) to 9 (*have not had sex in the last 12 months*), with an additional option of “*never had sex*”. We reverse coded these responses so that higher numbers indicated more frequent sexual behavior. Note that 907 participants reported that they had never had sex, and were thus excluded from this particular analysis.

## Study 1 results

Descriptive statistics and zero-order correlations are presented in Table 1.

**Table 1:** Zero order correlations and descriptive statistics.

	Zero-order correlations				
Variables	1	2	3	4	5
1. Age	—				
2. Gender	.12**	—			
3. Emoji use frequency	-.36**	.05**	—		
4. No. first dates	-.12**	-.07**	.19**	—	
5. Frequency of sex	-.30**	-.17**	.28**	.34**	—
Descriptive statistics					
*M*	42.03	1.56	4.63	1.56	3.39
*SD*	16.79	0.50	1.42	3.07	2.57

*M* = mean, *SD* = standard deviation.

**p < .01.

### Emoji/emoticon use

Use with potential dates was nuanced: 37.6% reported never using, and 29.1% reported hardly using, emojis/emoticons with potential dates. However, 28.2% regularly used emojis/emoticons with dates, 2.6% used at least one in every text to a date, and 2.5% used more than one in every text to a date. Participants’ motives for using emojis/emoticons with potential dates were that they give text messages more personality (53%), they make it easier to express feelings (23.5%), they are faster and easier than writing a full message (19.9%), and because they are trendy/are used by other people (12.5%).

### Emoji/emoticon use with dating and sex histories

We conducted two separate linear regressions—one for number of dates and one for sex frequency over the past year. Emoji/emoticon use served as the predictor variable. We also controlled for age and gender. All predictors were mean-centered. Regression statistics are presented in Table 2. Results indicated that participants who used emojis/emoticons more frequently went on more first dates, and engaged in sexual activity more often over the last year.

**Table 2:** Regression coefficients for the model predicting number of first dates and amount of sexual activity over the last year from frequency of emoji use, controlling for age and gender.

	Outcome variables							
	Number of first dates in the last year				Frequency of sexual behavior in the last year			
	*b*	95% CI	*t*_5322_	*r_p_^2^*	*b*	95% CI	*t*_4416_	*r_p_^2^*
Age	-0.01	[-0.017, -0.006]	-4.28***	-.06	-0.03	[-0.04, -0.03]	-12.75***	-.19
Gender	0.44	[0.28, 0.61]	5.29***	.07	0.84	[0.70, 0.99]	11.55***	.17
Emoji use with dates	0.52	[0.43, 0.61]	11.69***	.16	0.54	[0.46, 0.62]	13.50***	.20

Model 1: *R*^*2*^ = 0.05, *R*^*2*^ adjusted = 0.04, *F*(3, 5322) = 83.44, *p* < .001; Model 2: *R*^*2*^ = 0.15, *R*^*2*^ adjusted = 0.15, *F*(3, 4416) = 250.49, *p* < .001. *b* = unstandardized regression coefficient, *t* = *t* value, *r*_p_^2^ = partial eta squared.

***p < .001.

## Study 1 summary

Nearly 30% of this U.S. national sample of adult singles used emojis/emoticons regularly with dates, and most reported doing so because they provide a better outlet for self-expression than strictly text-based messages. Importantly, emoji/emoticon use was associated with more first dates and more frequent sexual activity over the last year.

## Study 2

We replicated and extended Study 1, investigating whether the frequency of singles’ emojis use with potential dates relates to success in intimate connections. We examined number of first dates and sexual frequency over the last year, second dates and further meet-ups, as well as details on their most recent date with which they used emojis: emoji frequency, intimate behaviors, and continued contact with this date.

## Study 2 method

### Participants

Participants were 275 single American adults (137 women; 136 men; 2 who choose not to report their gender identity). Age ranged from 18–71 years (*M* = 30.86, *SD* = 8.05). Most (83.6%) were exclusively or mostly heterosexual/straight, 13.5% were bisexual, and 3% were exclusively or mostly homosexual/gay/lesbian. All participants were single and not dating anyone (77.8%) or casually dating (22.2%). Most (72.7%) identified as White/Caucasian, 13.5% were Black/African-American, 8.7% Hispanic/Latino, 7.6% Asian, 1.5% Native American or American, 0.4% Arab or Middle Eastern, Indian, and 0.7% Biracial or multiethnic.

### Procedure

Data were collected in fall 2018. We recruited participants via Mechanical Turk. Inclusion criteria were being at least 18 years old, fluent in English, living in the U.S., not in a committed romantic relationship, and currently engaging in online dating via any app or website. Participants were also required to affirmatively respond to the item, “do you send or receive text messages, iMessages, Facebook messages, or other messages in which you could use emoji if you wanted to?” Participants completed demographics, items on their CMC preference and frequency, emoji use, romantic and sexual history over the past year, and details on their most recent first date. All data were collected over the Internet. Data access and analysis procedures were approved by Indiana University’s Institutional Review Board. Participants received the project description and a study information sheet prior to viewing the survey. They confirmed their consent via the online portal before partaking in the survey. Inclusion criteria required being at least 18 years old, fluent in English, and single (i.e., single and not seeing anyone or single but dating casually).

### Measures

#### Demographics

Participants reported their age, gender, ethnicity, level of education, sexual orientation, and relationship status. As in the previous study, only age and gender were included in analyses and only those identifying as men or women were included in the analyses (i.e., two participants were excluded).

#### CMC preference

Participants reported their agreement with the following: “Overall, I prefer to communicate with others through computer-mediated means, rather than face-to-face or speaking on the telephone” [26]. CMC was defined as “any kind of interactions with another person using a computer or smartphone that is connected to the Internet or a network connection (e.g., email, text message, app message, video chat).” Responses were 1 = *completely disagree*, 7 = *completely agree*.

#### CMC frequency

Participants were asked “How many of your daily interactions with others are computer mediated?” [26]. Responses were 1 = *none*, *0% of my daily interactions;* 7 = *all of the time*, *100% of my daily interactions*.

#### Emoji use

Emojis were defined as “small digital images or icons that can be inserted into text messages, iMessages, emails, or Facebook messages” followed by an example graphic of emojis. Participants responded to the item, “In text messages to potential dates, how often do you use emojis?” (1 = *never*, 7 = *very frequently*).

#### Number of first dates in the past year

Participants reported the number of first dates they had had over the past year (0–20+).

#### Number of second dates

Participants reported how many of the first dates they went on in the past year had led to a subsequent meet-up or second date (0–20+).

#### Sexual behavior in the past year

Participants reported how many sexual partners they had in the past year (open-ended) and how sexual frequency in the past year (1 = *haven’t had sex in the last year*, 8 = *multiple times a week*). Those (*n* = 57) who had never had sex were excluded from analyses.

#### Most recent first date

Participants reported when their last first date had occurred and whether they engaged in a text conversation prior to meeting in person. Only those who had texted with this date prior to meeting (*n* = 253; 92%) were included in the analyses of recent first date variables. They reported approximately how often they had used emojis with this person before the date (1 = *not at all*, 7 = *very frequently*), whether they saw this person again for a second date (*yes*, *no*, *not yet but we have plans to meet again*; recoded so that *yes* [*n* = 179] and *not yet but we have plans to meet again* [*n* = 13] were collapsed together), kissed (*yes*/*no*), had sex (*yes*/*no*), and/or entered into a relationship with this person (*yes*/*no*). Last, they reported how often they were in contact with this person at the time of the study (1 = *never*, 7 = *very frequently*).

## Study 2 results

Descriptive statistics and zero-order correlations are presented in Table 3.

**Table 3:** Zero-order correlations and descriptive statistics for all variables measured in Study 2.

Variables	1	2	3	4	5	6	7	8	9	10	11	12	13	14	15
1. Age	—														
2. Gender	0.03	—													
3. CMC preference	-0.15*	-0.18**	—												
4. CMC frequency	-0.10	0.01	0.41**	—											
5. Emoji freq^a^	-0.10	-0.25**	0.32**	0.23**	—										
**Behaviors in last year**														
6. # 1^st^ dates	0.04	-0.06	-0.07	-0.002	0.02	—									
7. # 2^nd^ dates	-0.01	-0.04	-.07	-0.001	0.15*	**	—								
8. Sex freq.	-0.00	0.10	-0.12	-0.07	0.13	0.37**	0.36**	—							
9. # sex partners	-0.09	0.11	-0.09	0.05	0.12	0.57**	0.64**	0.55**	—						
**With most recent first date**															
10. Emoji freq^b^	-0.10	-0.15*	0.31**	0.21**	0.62**	0.08	0.22**	0.07	0.15*	—					
11. Had 2^nd^ date	0.10	0.05	-0.01	-0.05	0.18**	0.001	0.15*	0.09	0.04	0.15*	—				
12. Kissed	0.11	0.09	0.04	0.02	0.24**	0.02	0.15*	0.27**	0.15*	0.19**	0.49**	—			
13. Had sex	0.09	0.13*	0.003	0.05	0.16*	0.02	0.17**	0.30**	0.22**	0.20**	0.34**	0.60**	—		
14. Entered relationship	0.05	-0.02	0.21**	0.08	0.25**	-0.12	0.11	0.01	0.02	0.35**	0.21**	0.36**	0.37**	—	
15. Current contact freq	0.03	-0.07	0.21**	0.05	0.40**	0.08	0.33**	0.25**	0.10	0.45**	0.35**	0.50**	0.41**	0.53**	—
**Descriptive statistics**															
*M*	30.86	0.50	4.82	56.69	5.11	4.41	2.81	3.83	2.48	4.71	0.83	0.73	0.55	0.33	4.04
*SD*	8.05	0.50	1.62	25.66	1.57	4.15	2.79	1.97	2.34	1.67	0.65	0.44	0.50	0.47	1.97

^a^Emoji use frequency with potential dates overall

^b^Emoji use frequency with most recent date

**p < .01

*p < .05.

### Emoji use with potential dates

Participants used emojis with potential dates quite frequently (*M* = 5.11, *SD* = 1.57), while only 3.3% reported that they never used emojis with potential dates.

### Emoji use and romantic and sexual behavior over the past year

To investigate whether more frequent emoji use with potential dates was associated with more first dates, second/follow-up dates, more frequent sexual activity, and more sexual partners over the past year, we conducted four separate linear regressions—one for each of these outcome variables. Frequency of emoji use served as the predictor variable. We also entered gender, age, CMC preference, and CMC frequency as control variables. We again did not test interactions to avoid increasing Type I error rate. All predictors were mean-centered. All regression coefficients for romantic and sexual behavior over the past year are presented in Table 4.

**Table 4:** Regression coefficients for four models predicting romantic and sexual behavior over the past year by emoji use frequency with potential partners in Study 2.

	How often do you use emojis?				
Predictor variables	*b*	95% CI		*t*	*r*_p_^2^
Model 1: *number of 1*^*st*^ *dates in past year*					
Age	0.004	[-0.06, 0.07]		0.12	0.01
Gender	-0.77	[-1.81, 0.27]		-1.46	-0.09
CMC preference	-0.27	[-0.63, 0.08]		-1.52	-0.09
CMC frequency	0.002	[-0.02, 0.02]		0.22	0.01
Emoji use frequency with potential partners	0.14	[-0.21, 0.49]		0.77	0.05
Model 2: *number of 2*^*nd*^*/follow-up dates*					
Age	0.00	[-0.04, 0.04]		-0.10	-.001
Gender	-0.02	[-0.72, 0.68]		-0.06	-0.004
CMC preference	0.04	[-0.20, 0.28]		0.34	0.02
CMC frequency	-0.003	[-0.02, 0.01]		-0.46	-0.03
Emoji use frequency with potential partners	0.27	[0.04, 0.51]		2.27*	0.14
Model 3: *sex frequency in past year*					
Age	0.002	[-0.03, 0.04]		0.15	0.01
Gender	0.37	[-0.17, 0.92]		1.35	0.09
CMC preference	-0.15	[-0.33, 0.04]		-1.58	-0.11
CMC frequency	-0.01	[-0.02, 0.01]		-0.98	-0.07
Emoji use frequency with potential partners	0.28	[0.10, 0.46]		3.05**	0.21
Model 4: *number of sex partners in past year*					
Age	-0.02	[-0.06, 0.02]		-1.17	-0.08
Gender	0.54	[-0.12, 1.20]		1.60	0.11
CMC preference	-0.20	[-0.42, 0.02]		-1.77	-0.12
CMC frequency	0.01	[-0.01, 0.02]		1.12	0.08
Emoji use frequency with potential partners	0.27	[0.05, 0.48]		2.43*	0.17

Model 1: *R*^2^ = 0.02, *R*^2^ adjusted = -0.001, *F*(5, 265) = 0.95, *p* = .45; Model 2: *R*^2^ = 0.03, *R*^2^ adjusted = 0.01, *F*(5, 264) = 1.37, *p* = .24; Model 3: *R*^2^ = 0.06, *R*^2^ adjusted = 0.04, *F*(5, 208) = 2.59, *p* < .05; Model 4: *R*^2^ = 0.06, *R*^2^ adjusted = 0.03, *F*(5, 205) = 2.47, *p* < .05. *b* = unstandardized regression coefficient, *t* = *t* value, *r*_p_ = partial correlation

**p < .01

*p < .05.

Frequency of emoji use was not significantly associated with number of first dates over the past year (*p* = .44). However, those who used emojis more often with potential dates reported a greater number of second/follow-up dates, more frequent sexual activity over the past year, and a greater number of sex partners over the past year.

### Emoji use and most recent date

We conducted five separate binary and linear regressions, depending on the type of outcome variable, which included the presence of a second/follow-up date with this person; more intimate behaviors with them, including kissing, having sex, and entering into a relationship together; and more frequent/current contact with that person. Frequency of emoji use served as the predictor variable. Participant gender, age, CMC preference, and CMC frequency were control variables. All predictors were mean-centered, and interactions were not examined.

Participants who used emoji more often were more likely to see their most recent date again for a second date, or have plans to do so (*OR* = 1.33 [1.10, 1.61], *p* < .01). They were also more likely to have kissed (*OR* = 1.38 [1.14, 1.68], *p* = .001), to have had sex (*OR* = 1.36 [1.14, 1.61], *p* = .001), and to have entered into a relationship with their most recent date (*OR* = 1.67 [1.35, 2.07], *p* < .001). Those who used emojis more often with their recent date also reported current, more frequent contact with that person (*b* = 0.51, *t*_244_ = 7.28, *p* < .001, *r*_p_ = .42).

## Study 2 summary

The majority of participants reported frequent emoji use with potential partners. Emoji frequency was unrelated to the number of first dates over the past year, but was related to maintaining connections with a first date (i.e., continued communication, second dates) and with more sexual behavior over the past year. Those who used emojis more with potential partners prior to the first date were more likely to have engaged in intimate behaviors with that person, and were more likely to have established a relationship with this person.

## General discussion

Drawing on affect, evolutionary, and social penetration theories, our study helps show that in studying emoji, we may better our understanding of human social psychology in the modern digital world. Affect, and both the production and reception of emotion, is fundamental to human experience, communication, and bonding [16]. Rooted in primary neurobiological systems for sociality, affect is an integral part of evolved human social behavior and human courtship. The ability and willingness to communicate affect and emotion provides important cues and signals that people use to assess a potential partner. Moreover, after initial interaction, affect becomes important for elements critical to interpersonal communication including relationship development and relationship maintenance, such as self-disclosure and vulnerability to a close relationship. Yet, in today’s digital era where increasing numbers of people are looking for romantic and sexual partners online and regularly communicating via CMC, the ability to effectively express and interpret emotions and valance can be difficult. The results of our studies suggest that emojis can thus be used, at times strategically, to imbue CMC with expression in ways that satisfy fundamental human desires for affect, intimate communication, and interpersonal closeness. In other words, we find that the use of emojis allows daters to communicate important affective information to potential partners which facilitates successful intimate connection and more romantic and sexual opportunities.

Across two studies, we examined the prevalence of emoji use with potential partners and related attitudes, motives for sending emojis to potential partners, and associations of emoji use with romantic and sexual relational experiences. Our samples indicated that emojis allowed for easier affective expression in CMC. Participants reported frequent use of emojis with potential romantic/sexual partners in both studies. Thus, it seems that emoji use has important implications for social interactions and connection, and this includes intimate connection.

In Study 1, we demonstrated that people who use emojis more often may be better at forming connections with others, as measured by the positive association between frequency of emoji use with potential partners and more dating and sexual behavior. In a large and diverse national sample of single adults, we found that participants who use emojis more often with a potential date also went on more dates over the last year. Although this effect was small and first dates do not necessarily result in a long-term relationship, these participants were successful at the initial courtship stages of cultivating shared interest and gaining the opportunity to try and build the foundation for a deeper intimate connection. Relatedly, those who used emojis more often with potential dates also reported more sexual activity over the last year. As with first dates, we cannot assume that sexual engagement led to further interaction. However, these participants were successful at building connections involving attraction, chemistry, and comfort—essential components for engaging in physical intimacy. That is, in both cases of dating and sexual frequency, this suggests that emoji users are also engaging psychological courtship systems in their social interactions, and doing so with some degree of success. These users are demonstrating that they are more emotive across the affect spectrum, which leads to more effective social interactions and interpersonal communication, and is associated with more romantic and sexual opportunities.

In Study 2, we replicated the findings of Study 1, showing that participants were frequently sending emojis to potential partners, with only 3.3% of the sample reporting that they never use emojis in this context. Although the finding from Study 1 that emoji use frequency was related to more first dates did not replicate in Study 2, we found that emoji use with potential partners was associated with having a second date or meet-up with a potential partner, more frequent sexual activity, and more sexual partners over the past year. Again, this suggests that those who use emojis more often in this context are more successful at establishing connection and thus enjoy more opportunities for romantic and sexual engagement.

### Limitations and future directions

There are limitations to our studies that future research should address. First, because all studies were correlational and only assessed sending emojis, we cannot know how the recipient perceives emoji-laden messages, or how emoji interpretation differs depending on individual characteristics of the recipient. Additionally, because certain traits can influence how communication partners interpret the contents of their CMC with each other [27], future research would benefit from conducting additional dyadic research to answer these inquiries.

In addition, our studies do not allow for assessing causality. We cannot determine whether more emoji use leads to more dates and sex or vice-versa, however, it is clear that there is a significant association. It is possible that all these factors are products of more global underlying neurobiological affective systems, similarly resulting from shared process systems for affect and social behavior. While we might speculate that emoji use is an extended feature of those with traits that have been linked to better quality relationships, such as greater levels of emotional intelligence [28], future longitudinal studies will be able to more precisely answer this question.

We did not assess the specific emojis sent to potential partners, the total numbers of which are quite vast. Thus, we cannot fully know which emojis are most effective at helping to form connections between people. Similarly, the finer morphological features of specific emojis—such as facial expression, shape of eye and mouth, presence of teeth, colors, etc.—may make some emojis more effective at communicating affect than others. For instance, a recent study demonstrated that individuals preferentially use specific types of emoji with specific recipients, such as facial expressions for emotion and objects resembling body parts, to communicate sexually suggestive messages and initiate sexting [29]. Further, while the current study focused on English-speaking participants, these findings may vary across languages and cultures. These questions should be further explored in future studies.

Future work may also use emojis to further connect research in social psychology, communications, and relationship science. Researchers continue to uncover the role of communication in people’s romantic and sexual lives [30, 31], and this extends to the importance of non-verbal behaviors in relationships, such as affection and positive affect [32]. Indeed, positive text messages to a partner have been associated with increased relationship satisfaction [33]. Similarly, sexting may also play a role in establishing and maintaining romantic and sexual relationships [34]. How these various non-verbal behaviors are communicated and interpreted, and whether they offer the same benefits in CMC, remains an open question. Use of these images may have farther reaching impacts on people’s romantic and sexual relationships, such as initiating (digital) sexual conversations or maintaining connection and intimacy [35].

### Conclusion

In sum, we examined frequency of emoji use with potential partners, attitudes surrounding emoji use with potential partners, motives for using emojis with those partners, and whether such use relates to more opportunities to form romantic and sexual connections. Combining findings from two different studies on adult singles, our research suggests that emoji use may provide a reasonable proxy for expressing affect and may provide a useful aid in self-disclosure and building intimacy. As a result, using emojis with potential romantic and sexual partners may in turn lead to more face-to-face opportunities to assess compatibility and attraction. Emojis appear to be an important aspect of social behavior in today’s digital world that can be used strategically as affective signals, particularly in the domain of human courtship.

## Supporting information

S1 File Study1Dataset.6.24.2019. SPSS data file containing all variables used in Study 1.
(SAV)

S2 File Study2Dataset.6.24.2019. SPSS data file containing all variables used in Study 2.
(SAV)
